# How Wealth Inequality Affects Happiness: The Perspective of Social Comparison

**DOI:** 10.3389/fpsyg.2022.829707

**Published:** 2022-04-11

**Authors:** Lingxi Gao, Bochi Sun, Ziqing Du, Guangming Lv

**Affiliations:** School of Statistics, Beijing Normal University, Beijing, China

**Keywords:** happiness, social comparison, wealth inequality, Gini coefficient, the upward wealth inequality, the downward wealth inequality, life cycle

## Abstract

Since Easterlin pointed out that economic growth in nations does not guarantee increasing happiness for the average citizen, the underlying reason has remained controversial. The present study focuses on income inequality to explain the “Easterlin Paradox,” ignoring the permanent inequality that long-term wealth accumulation brings. Based on social comparison theory, the literature aims to determine how wealth inequality, which accompanies economic growth, diminishes one’s happiness (inequality aversion). Specifically, we conduct this study in which we split the wealth inequality into the upward wealth inequality and the downward wealth inequality as measures of upward comparison and downward comparison, respectively. The upward wealth inequality measures the average gap between one and the better-off in wealth while the downward wealth inequality measures the average gap between one and the worse-off in wealth. Furthermore, the heterogeneity of the area of respondent is analyzed and the family life cycle is tested as a moderator. The main findings of the paper are as follows: (1) The empirical test results of hypothesis 1 indicate that the upward wealth inequality aversion (jealousy effect: people envy who is richer than themselves) is stronger than the downward wealth inequality inclination (proud effect: people enjoy having a superior position in the wealth hierarchy). It is due to the psychological preference: loss aversion. As an increase in upward distance implies a loss in relative status and an increase in downward distance implies a gain in relative status, people focus more on loss rather than gain. (2) The empirical test results of hypothesis 2 indicate that residents who live in rural areas do not have a proud effect as much as those who live in urban areas. There is a huge urban-rural wealth gap in China. With the expansion of the social network, people living in rural areas realize that even he is almost the rich in rural areas but still the lower classes in the whole society. It is hard for rural residents to have a proud effect. (3) The empirical test results of hypothesis 3 indicate that family members have the strongest upward inequality aversion in the middle-stage phase of the life cycle (when the family head is approximately 50). During the family life cycle, inequality aversion will be different in different life stages due to the changes in economic status expectations. At the beginning of the family life cycle, family members assume their life has limitless possibilities, and they have high expectations for the future. Logically, they can be easily satisfied by achieving a little more than their peers. In later periods, with increasing age, the members will pay more attention to health instead of wealth. The results shed light on how macroeconomics influence changes in individual psychology.

## Introduction

There is a long history of debating the causes of “Easterlin Paradox”. The “Easterlin Paradox” suggests that a positive relationship between inequality and happiness does not exist (Easterlin, 1974). The present paper is situated at the intersection of two lines of research. One is research based on the limits of economic growth. This literature attempts to prove that it is nonmaterial factors that jeopardize the average citizen’s subjective well-being and that they offset the positive subjective well-being effect of material factors, for example, air pollution (Zhang et al., 2017), traffic congestion (Smyth et al., 2008), crime (Powdthavee, 2005), etc. In particular, economic growth is inevitably paired with economic inequality, which could lead to undermining social cohesion and residential segregation (Bjorvatn and Cappelen, 2003; Engler and Weisstanner, 2021).

The second line of the research explains the social comparison that arises with inequality. Based on social comparison theory (Festinger, 1954), one’s relative economic position has a significant effect on one’s happiness. Poorer people could be happier than richer people if they are relatively rich in their surroundings. Furthermore, economic inequality triggers people’s awareness of their economic status, and those with lower economic status tend to perceive relative deprivation. The more deprived one feels, the unhappier one becomes (D’Ambrosio and Frick, 2007). Apart from the horizontal social comparison (compared to individuals’ surroundings), the vertical social comparison (compared to an individual’s wealth) explains the way happiness does not catch up with economic growth. It could be the result of hedonic adaptation through vertical social comparison. Researchers have found that people recover after sudden shocks in their income (Di Tella and MacCulloch, 2010; Di Tella et al., 2010). Thus, the level of happiness returns to normal after the economic shock.

The social comparison theory categorized the horizontal social comparison into the upward horizontal social comparison and downward horizontal social comparison (Gibbons and Gerrard, 1989; Gerber et al., 2018). Upward horizontal social comparison is defined as comparing with better-off others while the downward horizontal social comparison is defined as comparing with worse-off others. People constantly evaluate themselves, especially in economic domains like income, wealth, consumption, etc. Thus, people are not only concerned about their absolute wealth but also their relative wealth by comparing with the better-off and the worse-off. Fehr and Schmidt (1999) take the initiative in qualifying the upward economic distance (distance between individual and better-off others) and downward economic distance (distance between an individual and worse-off others) as upward inequality and downward inequality. They also posit four conditional forms of people’s affection towards them. An individual is envious if he feels deprived by the high upward inequality while the “tunnel effect” (perceiving the opportunity to keep up with the rich) is active if he is happy about it. In the meantime, an individual is proud if he feels happy about the high downward inequality while he is altruistic if he feels upset about it, Researchers verify those affections empirically (Hopkins, 2008; Espín et al., 2018). Yu and Wang (2017) compare two conflicting effects, the signal effect and jealousy effect, in the relationship between inequality and happiness. They report that the happiness-inequality relationship is an inverted U-shaped curve that peaks at the signal effect and is then offset by the jealousy effect. As the inequality increases, the jealousy effect gradually overcomes the signal effect.

Oishi et al. (2011) explain how inequality harms happiness. He claims that inequality lowers perceived fairness and general trust, thereby upsetting people. Previous studies assume that an uneven income distribution always has a negative effect on happiness. However, there are a few studies on the positive relationship between inequality and happiness. Hirschman and Rothschild (1973) suggest that inequality could be interpreted as a positive signal for the poor since it indicates a promising future. The phenomenon is known as the “tunnel effect” and it suggests social mobility may moderate the effect of inequality on happiness. A person may see others’ upward mobility as positive as it can provide a positive signal about himself when the level of social mobility is high. There are other moderators that affect the relationship between inequality and happiness. These moderation findings extend our understanding of the way that different moderators can explain the different results of how inequality affects happiness in empirical studies. Many empirical findings suggest that the sense of fairness plays an important role in the link between inequality and happiness (Shaw and Olson, 2014; Starmans et al., 2017). People are quite tolerable of inequality that is perceived as a result of a fair allocation mechanism. Ugur (2021) suggests that individuals’ perceptions of fairness primarily affect inequality aversion. The underlying reason might be the perceived unfairness increases as the inequality increases and that influences the process of how inequality affects happiness. In addition, Ding et al. (2021) highlight the importance of urban-rural heterogeneity in China for inequality aversion.

In the field of happiness research, happiness varies with age in U-shape has been proved by a lot of papers (Blanchflower and Oswald, 2004, 2008; Blanchflower, 2021). Stone et al. (2010) explain it with socioemotional selective theory: the elderly are more effective at regulating their emotions positively than young adults. Older people view their situation positively and the level of their happiness increases.

The existing literature usually focuses on income inequality’s effect on subjective well-being. However, income inequality is more likely to be transitory than permanent. Permanent inequality could lead to a more marked reduction in happiness than transitory wealth inequality. Since household wealth returns tend to be strongly persistent (Bach et al., 2020), it is easier for the rich to get richer and harder for the poor to escape the poverty trap. Thus, those who do not have enough initial endowment might feel unfairly affected.

Based on the literature reviewed, we next propose three hypotheses: hypothesis 1: wealth inequality would have a significant effect on happiness; hypothesis 2: the effect of wealth inequality on happiness would exhibit urban-rural heterogeneity; hypothesis 3: wealth inequality would affect happiness differently in different family life stages.

We contribute to the existing literature by examining the link between split wealth inequality and happiness. Based on the social comparison theory, the Gini index are decomposed into the upward wealth inequality and the downward wealth inequality, and the indexes measures the different economic gap when families compare with different target. The perceived wealth distance for families with both the better-off and the worse-off helps them to complete self-evaluation and social comparison. The wealth Gini coefficient measures the regional inequality and can be defined as the average value across families of personal inequality index in this region. The decomposing wealth Gini coefficient into upward wealth inequality and downward wealth inequality enables us to decompose the regional wealth inequality into personal wealth gap including the average gap between one and the better-off, or worse-off. The upward wealth inequality represents deprivation with respect to being better off while the downward wealth inequality represents advantage with respect to being worse off. The regression of upward wealth inequality and downward wealth inequality makes it possible to capture the families’ affections, respectively. It turns out the upward wealth inequality aversion (jealousy effect: people envy who is richer than themselves) is stronger than downward wealth inequality inclination (proud effect: people enjoy having a superior position in the wealth hierarchy) in general. The remainder of the paper evaluates the inequality aversion model based on a large dataset, verifies the moderating effects of the life cycle and examines the urban-rural heterogeneity of baseline regression. It shows family members have the strongest upward inequality aversion in the middle-stage phase of the life cycle.

## Empirical Test of Hypothesis 1: The Regression of Wealth Inequality to Happiness

In the empirical test of hypothesis 1, we examine the effect of wealth inequality on happiness and how the preference for inequality driven by the status between the self and relevant others can lead to two opposite effects. The Gini index measures the total inequality effect but is unable to capture the upward wealth inequality and the downward wealth inequality. Hence, we further divide the wealth inequality into upward relative concern and downward relative concern based on the social comparison thesis. Then, we examine the relationship between relative concern in different directions and happiness. In fact, the upward wealth inequality measures the distance gap between one and one’s richer peers. The “keeping up with the Joneses” effect implies that the larger the relative gap between one and one’s richer peers is, the unhappier one is. The effect of an increase in the wealth of one’s richer peers on one’s happiness is negative since one will envy them (Friedman and Ostrov, 2008).

### Sample

Data that support the empirical analysis come from the China Household Finance Survey (CHFS) conducted by the Southwestern University of Finance and Economics in China. The survey has been conducted every two years since 2011. Since the 2011 sample is the beta version and the 2013 sample have less respondents, we use the sample included in 2015 and 2017. The total sample combines of 2015 and 2017 has 52,348 individuals. All households are randomly selected from 29 provinces in China. From 2015 to 2017, the survey covers 1,396 and 1,428 municipalities nationwide respectively, which is a representative sample of China. We merge the household sample and individual sample to track the fixed households and create cross-sectional data with 2015 and 2017 sample.

The data include family assets, happiness, and sociodemographic characteristics. Thus, the data allow us to further include economic characteristics that significantly explain happiness.

### Measures

#### Happiness

Happiness is defined as a subjective evaluation of one’s life-as-a-whole. The variable happiness is extracted from the following question: “In general, do you feel like happy now?”. The answers are classified as (1) Very unhappy, (2) Unhappy, (3) Generally, (4) Happy, and (5) Very happy. Happiness is rated by the respondent on a five-point rating scale. We construct a five-point scale (5 = Very happy, 4 = Happy, 3 = Generally, 2 = Unhappy, 1 = Very unhappy) to measure happiness. In this paper, we concentrate on the family, thus, the family head’s happiness is representative of the family’s happiness since the family head is usually the sole decider and emotion can be contagious inside the family.

Fowler and Christakis (2008) prove the spread of happiness inside social networks, such as the family. Thus, the subjective well-being of family members is similar. Happiness is measured at the ordinal level.

#### Wealth Inequality

In this paper, we concentrate on inequality that increases permanent inequality. We use wealth inequality to capture permanent inequality since it is relatively stable. Based on social comparison as a consistent psychological phenomenon, we attempt to explain how permanent inequality affects happiness.

The wealth Gini coefficient of the population is used to compare wealth inequality across populations. In fact, it can be interpreted as the mean of personal inequality. However, the Gini coefficient is a measure of the wealth distribution of a region’s household, and it uses the sum of all pairwise absolute wealth differences.


(1) $$\text{G}=\frac{1}{2\,{\text{n}}^{2}\,\bar{\text{y}}}\,\sum_{i=1}^{n}\sum_{j=1}^{n}\left|{\text{y}}_{j}-{\text{y}}_{i}\right|$$

where *y_i* and *y_j* is the wealth of household *i* and *j* respectively, $\bar{y}$ is the mean wealth, *n* > 1, and y1≤*y*_2_≤⋯≤*y*_*n*_.

Thus, even though the Gini coefficient is a widely used index for inequality, it cannot be used as the basis for individual or household inequality. To focus on the relationship between household happiness and the inequality among families, we measure inequality at the household level. We regard household inequality from a “top down” or “bottom up” perspective.

#### The Upward Wealth Inequality and the Downward Wealth Inequality

Based on the easily overlooked fact that the Gini coefficient is the mean value across the household of a particular region:


(2) $$\text{G}=\frac{1}{\text{n}}\,\sum_{i=1}^{n}{\text{G}}_{i}$$

where *G_i* is the average absolute difference between household *i* and peers in their reference group:


(3) $${\text{G}}_{i}=\frac{1}{2\,\text{n}\,\bar{\text{y}}}\,\sum_{j=1}^{n}\left|{\text{y}}_{i}-{\text{y}}_{j}\right|$$

*G_i* can be rewritten as:


(4) $${\text{G}}_{i}=\frac{1}{2\,\text{n}\,\bar{\text{y}}}\,\left[{\text{n}}_{i}^{l}\,\left({\text{y}}_{i}-{\bar{\text{y}}}_{i}^{l}\right)+{\text{n}}_{i}^{h}\,\left({\bar{\text{y}}}_{i}^{h}-{\text{y}}_{i}\right)\right]$$

Where $n_{i}^{l}$ is the number of households with wealth less than or equal to *y_i* and $n_{i}^{h}$ is the number of households with wealth greater than *y_i*. Excluding household *i*, $n_{i}^{l}+n_{i}^{h}=n-1$. Thus, *G_i* can be split into the upward wealth inequality and the downward wealth inequality.


(5) $${\text{up}}_{i}=\frac{1}{2\,\text{n}\,\bar{\text{y}}}\,{\text{n}}_{i}^{h}\,\left({\bar{\text{y}}}_{i}^{h}-{\text{y}}_{i}\right)$$

(6) $${\text{down}}_{i}=\frac{1}{2\,\text{n}\,\bar{}}\,{\text{n}}_{i}^{l}\,\left({\text{y}}_{i}-{\bar{\text{y}}}_{i}^{l}\right)$$

The upward wealth inequality represents deprivation with respect to being better off. The incessant perception of relative deprivation and positional concern can be a source of envy in the reference group (Sultana, 2019). Feelings of loss and envy negatively affect happiness. The downward wealth inequality represents an advantage with respect to being worse off. Some researchers find fair and cooperative behavior in experimental studies, such as ultimatum games (Thaler, 1988; Nowak et al., 2000) or gift exchange games (Fehr et al., 1997; Charness et al., 2004). We believe that the measures of the upward wealth inequality and the downward wealth inequality could capture aversion to inequality from different perspectives.

### Control Variables

We consider the relevant variables that influence family happiness. Those variables include both demographic characteristics of the family head and family, such as age of the family head, education of the family head, marital status of the family head, gender of the family head, work status of the family head, youth ratio, elderly ratio, pension of family members, family size, average education level of the family and whether they have their own houses. These variables reflect the economic and demographic situation of the family head and family. All variables used in this study are summarized in Table 1.

**TABLE 1:** Variables definition.

Variable	Definition
**Dependent Variable**	
Happiness	Very happy, Happy, Generally, Unhappy, and Very unhappy (with values of 5 to 1)
**Independent Variable**	
The downward wealth inequality	Detail calculation refer to Equation (6)
The upward wealth inequality	Detail calculation refer to Equation (5)

**Family covariate**	
Youth ratio	The ratio of the youth (age ≤ 16) in family
Elderly ratio	The ratio of the elderly (age > 60) in family
Family’s pension	If any member in the family has pension: the value of 1; otherwise: the value of 0
Family’s medicare	If any member in the family has medical care: the value of 1; otherwise: the value of 0
Family size	The number of family members
Mean of family education	The average years of schooling in family
Whether own house property	If any members own a house property in the family: the value of 1; otherwise: the value of 0

**Family head Covariate**	
Family head’s age	The age of family head
Family head’s education level	The years of schooling for family’s head
Family head’s marital status	The family head is married: the value of 1; otherwise: the value of 0
Family head’s gender	The family head is male: the value of 1; the family head is female: the value of 0
Family head’s work status: Government official	The family head works as government official: the value of 1; otherwise: the value of 0
Family head’s work status: State-owned enterprises	The family head is engaged in the state-owned enterprise: the value of 1; otherwise: the value of 0
Family head’s work status: Private sector	The family head is engaged in the private sector: the value of 1; otherwise: the value of 0
Family head’s work status: Agriculture	The family head is engaged in agriculture: the value of 1; otherwise: the value of 0

### The Choice of Reference Group

The similarity criterion for social influence shows that individuals are most influenced by others who are similar. Considering the geographical factor, the basic premise of our model is a reference group with which one is most likely to compare. We established a reference group comprising individuals in the same community.

### Model Specifications

We assume the relative wealth hypothesis that the family cares about the relative wealth level as well as absolute wealth itself.


(7) $${\text{happiness}}_{\mathrm{ij}}=\alpha_{0}+\alpha_{1}\,\text{ln}\,\left({\text{wealth}}_{\mathrm{ij}}\right)+\alpha \cdot {\text{gini}}_{j}+\theta \cdot {\text{X}}_{\mathrm{ij}}+\varepsilon_{\mathrm{ij}}$$

(8) $${\text{happiness}}_{\mathrm{ij}}=\alpha_{0}+\alpha_{1}\,\text{ln}\,\left({\text{wealth}}_{\mathrm{ij}}\right)+\alpha \cdot {\text{up}}_{\mathrm{ij}}+\beta \cdot {\text{down}}_{\mathrm{ij}}+\theta \cdot {\text{X}}_{\mathrm{ij}}+\varepsilon_{\mathrm{ij}}$$

Where *happiness*_*ij*_ is latent happiness of household *i* in community *j*, *wealth*_*ij*_ is wealth of household *i* in community *j*, *gini_j* is the Gini index in community *j*, *up*_*ij*_ is the upward wealth inequality of household *i* in community *j*, *down*_*ij*_ is the downward wealth inequality of household *i* in community *j*, *X*_*ij*_ is the vector of control variables, ε_*ij*_ is error item follows normal distribution.

The measurements of happiness, as noted above, are five discrete responses. The discrete variable is difficult to capture the subtle different within the same category since five categories capture a latent continuum. The observed variable happiness *happiness*_*ij*_ will be determined based on the latent variable $h\,a\,p\,p\,i\,n\,e\,s\,s_{i\,j}^{*}$.

We cannot observe the actual level of family happiness, which lies in an ordered categorical range. Some researchers tend to use the ordered response model instead of the ordinary linear model to specify the distribution of the latent variable and avoid arbitrariness of the ordinal scale. Although happiness is always measured as a discrete variable in the survey, sometimes, a continuous latent variable is postulated, which is mapped onto the discrete measurement.

Most studies measure happiness using an ordered model rather than ordinary linear square regression (OLS regression). However, Ferrer-i-Carbonell and Frijters (2004) argue that the OLS regression or discrete response model makes little difference to the results. The ordered probit estimate is not suitable for explaining the average marginal effect on the latent variable. Since OLS regression makes it easier to interpret, in this paper, we use OLS regression to capture inequality aversion.

### Results and Discussion

Table 2 reports some basic statistics in our sample.

**TABLE 2:** Descriptive statistics.

	(1)	(2)	(3)
Variables	N	mean	SD
Gini index	52,348	0.508	0.119
happiness	52,348	3.806	0.838
The downward wealth inequality	52,348	0.256	0.526
The upward wealth inequality	52,348	0.253	0.152
Youth ratio	52,348	0.084	0.149
Elderly ratio	52,348	0.322	0.395
Family pension	52,348	0.882	0.322
Family medicare	52,348	0.961	0.193
Family size	52,348	3.130	1.478
Mean of family education	52,348	6.110	4.616
Whether own house property	52,348	0.913	0.282
Family head’s age	52,348	55.190	14.030
Family head’s education level	52,348	9.150	4.139
Family head’s marital status	52,348	0.859	0.348
Family head’s gender	52,348	0.794	0.404
Family head’s work status: government official	52,348	0.068	0.252
Family head’s work status: state-owned enterprises	52,348	0.052	0.222
Family head’s work status: private sector	52,348	0.233	0.423
Family head’s work status: agriculture	52,348	0.192	0.394
			

The regression starts by taking the total effect of wealth inequality into account. The model in Table 2 is the empirical test of permanent inequality aversion. In this study, we estimate inequality aversion based on pooled data. First, the wealth Gini coefficient has a significantly negative effect on happiness (b = −0.082, *p* < 0.001), which means that one standard increase in the Gini index in the surroundings decreases the proportion of people reporting themselves as “Very happy” by 0.20 percentage points. Overall, permanent inequality is negatively related to happiness, which means that the persistent gap between people upsets them. To clarify the mechanism of comparison-driven inequality aversion, we decompose the wealth Gini index into the upward wealth inequality and the downward wealth inequality.

Table 3 shows that based on the results of the regression of the upward wealth inequality and the downward wealth inequality on happiness, the upward wealth inequality has a significantly negative effect on happiness (b = -0.453, *p* < 0.001), while downward inequality has the opposite effect (b = 0.021, *p* < 0.05).

**TABLE 3:** OLS regression on the relationship between happiness and wealth inequality.

Gini Index				

Happiness	b	SE	t	*p*
**Independent variable**				
Gini index	–0.082	0.031	–2.600	0.009
**Family covariate**				
Youth ratio	0.470	0.036	12.990	0.000
Elderly ratio	0.445	0.024	18.600	0.000
Family’s pension	0.090	0.012	7.680	0.000
Family’s medicare	0.060	0.019	3.130	0.002
Family size	0.000	0.003	–0.050	0.964
Mean of family education	0.020	0.002	9.880	0.000
Whether own house property	0.080	0.013	6.060	0.000
**Family head’s covariate**				
Family head’s age	0.005	0.000	12.210	0.000
Family head’s education level	0.002	0.001	1.660	0.098
Family head’s marital status	0.183	0.012	15.880	0.000
Family head’s gender	–0.006	0.010	–0.610	0.540
Family head’s work status: government official	0.112	0.016	6.900	0.000
Family head’s work status: state-owned enterprises	0.053	0.018	2.950	0.003
Family head’s work status: private sector	0.086	0.011	8.150	0.000
Family head’s work status: agriculture	0.001	0.010	0.090	0.926

**Decompose Gini Index into the Upward Wealth Inequality and the Downward Wealth Inequality**				

**Independent variable**				
the Downward wealth inequality	0.021	0.008	2.540	0.011
the Upward wealth inequality	–0.453	0.032	–14.180	0.000
**Family covariate**				
Youth ratio	0.470	0.036	13.030	0.000
Elderly ratio	0.437	0.024	18.310	0.000
Family’s pension	0.079	0.012	6.760	0.000
Family’s medicare	0.049	0.019	2.570	0.010
Family size	–0.006	0.003	–1.950	0.051
Mean of family education	0.018	0.002	8.820	0.000
Whether own house property	–0.003	0.014	–0.240	0.812
**Family head’s covariate**				
Family head’s age	0.005	0.000	12.270	0.000
Family head’s education level	–0.001	0.001	–0.530	0.593
Family head’s marital status	0.172	0.011	14.960	0.000
Family head’s gender	–0.006	0.010	–0.650	0.516
Family head’s work status: government official	0.108	0.016	6.720	0.000
Family head’s work status: state-owned enterprises	0.048	0.018	2.680	0.007
Family head’s work status: private sector	0.086	0.011	8.120	0.000
Family head’s work status: agriculture	0.005	0.010	0.490	0.625
				

The results show an aversion to the upward wealth inequality and an inclination to the downward wealth inequality. However, the two components that the Gini index decomposes display asymmetry: the upward wealth inequality has a greater impact on happiness than the downward wealth inequality does. This trend mirrors people’s psychological preferences, as they would tend to focus on the increases among the rich and neglect decreases among the poor (Boyce et al., 2010).

The inequality aversion mainly comes from the upward wealth inequality, rather than the downward wealth inequality. The empirical results show that a persistent upward distance makes one feel worse and that a persistent downward distance makes one feel better. Compared to the result in temporary advantage that Cojocaru (2014) reveals, the permanent advantage makes status concerns exceed empathy. The temporary advantage arouses unfairness of the downward distance. In contrast, the larger one’s permanent downward distance is, the better one would feel. Additionally, the results show that upward inequality aversion is stronger than downward inequality aversion. The behavior concept behind this outcome might be loss aversion, and the upward wealth inequality can be seen as loss from others in the reference group. Households confirm their status quo from the social comparison. Thus, an increase in upward distance implies a loss in relative status, and an increase in downward distance implies a gain in relative status.

## Empirical Test of Hypothesis 2: Urban-Rural Heterogeneity of Inequality’s Effect on Happiness

Like many developing countries, the urban-oriented policies in China have contributed to the increasing urban-rural inequality. In particular, there are many aspects that contribute to it: governmental regulation of the price of agricultural products; the segmentation of urban and rural markets; and superior social welfare and social security of city residents. Given the dual-track of urban-rural structure, the residence could affect people’s affection towards wealth inequality.

### Results and Discussion

Based on Equations 7, 8, Considering the dual-track of urban-rural development, the influence of wealth inequality on happiness is next analyzed by the residence of families. The sample is categorized into two: families live in urban areas and families live in rural areas. As shown in Table 4, the upward wealth inequality increases happiness significantly among families living within both urban areas (b = -0.442, *p* < 0.001) and rural areas (b = -0.474, *p* < 0.001). the downward wealth inequality still decreases happiness significantly among families living in urban areas (b = 0.026, *p* < 0.05) as the baseline, but for families living in rural areas, the downward wealth inequality does not have a significant impact on happiness.

**TABLE 4:** Regression on happiness with the upward wealth inequality and the downward wealth inequality for stratified sample by residence.

Happiness	b	SE	t	p
**Independent Variable**				
**The downward wealth inequality**				
Family live in rural areas	0.015	0.015	1.020	0.309
Family live in urban areas	0.026	0.010	2.550	0.011
**The upward wealth inequality**				
Family live in rural areas	–0.474	0.057	–8.300	0.000
Family live in urban areas	–0.442	0.039	–11.320	0.000

**Family covariate**				
**Youth ratio**				
Family live in rural areas	0.599	0.065	9.150	0.000
Family live in urban areas	0.387	0.045	8.630	0.000
**Elderly ratio**				
Family live in rural areas	0.475	0.041	11.660	0.000
Family live in urban areas	0.405	0.031	12.960	0.000
**Family’s pension**				
Family live in rural areas	0.104	0.019	5.340	0.000
Family live in urban areas	0.063	0.015	4.280	0.000
**Family’s medicare**				
Family live in rural areas	0.026	0.035	0.760	0.445
Family live in urban areas	0.064	0.023	2.840	0.005
**Family size**				
Family live in rural areas	–0.008	0.005	–1.570	0.116
Family live in urban areas	–0.006	0.004	–1.450	0.147
**Mean of family education**				
Family live in rural areas	0.023	0.004	5.610	0.000
Family live in urban areas	0.015	0.003	5.710	0.000
**Whether own house property**				
Family live in rural areas	–0.029	0.035	–0.840	0.399
Family live in urban areas	0.011	0.015	0.710	0.477

**Family head Covariate**				
**Family head’s age**				
Family live in rural areas	0.006	0.001	7.340	0.000
Family live in urban areas	0.004	0.000	9.320	0.000
**Family head’s education level**				
Family live in rural areas	0.001	0.002	0.580	0.562
Family live in urban areas	–0.003	0.002	–1.790	0.073
**Family head’s marital status**				
Family live in rural areas	0.182	0.022	8.140	0.000
Family live in urban areas	0.165	0.013	12.450	0.000
**Family head’s gender**				
Family live in rural areas	0.056	0.023	2.470	0.014
Family live in urban areas	–0.020	0.011	–1.940	0.053
**Family head’s work status: Government official**				
Family live in rural areas	0.115	0.046	2.500	0.012
Family live in urban areas	0.116	0.017	6.820	0.000
**Family head’s work status: State-owned enterprises**				
Family live in rural areas	0.067	0.060	1.110	0.268
Family live in urban areas	0.053	0.018	2.850	0.004
**Family head’s work status: Private sector**				
Family live in rural areas	0.076	0.021	3.600	0.000
Family live in urban areas	0.088	0.012	7.260	0.000
**Family head’s work status: Agriculture**				
Family live in rural areas	0.007	0.015	0.470	0.637
Family live in urban areas	0.051	0.019	2.690	0.007
				

With the expansion of the social networks, people can easily obtain the information of others living far away. The huge urban-rural wealth gap caused by house price, labor market return, industrial structure, etc. is apparent. People come to realize that even he is almost the rich in rural areas but still has the lower classes in the overall society. This situation could deter them from being satisfied with their tiny achievement that they are richer in rural areas. Residents in rural area do not perceive the opportunity to narrow the gap to ascend in the social scale, which leads them to manifest non-significant pride for themselves.

## Empirical Test of Hypothesis 3: The Moderating Effects of Life Cycle

We further explore the effects of the life cycle on the relationship between wealth inequality and happiness. Since the family head is representative of the family, the family head’ age could reflect the life stage of the family. Then, we explore whether the family life cycle generates a moderating effect on the relationship between wealth inequality and happiness. A lot of literature has shown that in different life stages, the state of mind is different. Some researches prove that the relationship between age and happiness exhibits a U-shaped curve (Blanchflower and Oswald, 2008; Yu and Wang, 2017). People’s expectation plays an important role in economic decision and affection (Schadner and Schadner, 2021). Schwandt (2016) finds that the U-shaped relationship is driven by the unmet aspiration that makes one feel painful in his midlife. At the beginning of the family, members are excited about the promising future, but expectations might urge them to overestimate future satisfaction. Thus, in the middle stage of the family, family members might feel upset about the unrealized economic status goal. As time passes, in later stages, they gradually lower their expectations.

### Household Life Cycle

Since the family is a complete economic unit in society, the evolution of the family has been studied. Murphy and Staples (1979) conclude stages in the family life cycle as young stages, middle-aged stages, and older stages. Additionally, they mention that the family life cycle can be decided by the family head’s age. Considering that the head of the household makes the family decision and is representative of the family, we use the family head’ age as the proxy of the family life cycle.

### Model Specifications

Based on the baseline Equations 7, 8, we consider the moderating effect of the family life cycle. Moderation is typically tested through regression analysis.


(9) $${\text{happiness}}_{\mathrm{ij}}=\alpha_{0}+\alpha_{1}\,\text{ln}\,\left({\text{wealth}}_{\mathrm{ij}}\right)+\alpha \cdot {\text{gini}}_{j}+\beta \cdot {\text{gini}}_{j}\times {\text{hage}}_{\mathrm{ij}}+\theta \cdot {\text{X}}_{\mathrm{ij}}+\varepsilon_{\mathrm{ij}}$$

(10) $${\text{happiness}}_{\mathrm{ij}}=\alpha_{0}+\alpha_{1}\,\text{ln}\,\left({\text{wealth}}_{\mathrm{ij}}\right)+\alpha \cdot {\text{up}}_{\mathrm{ij}}+\beta \cdot {\text{down}}_{\mathrm{ij}}+\gamma \cdot {\text{up}}_{\mathrm{ij}}\times {\text{hage}}_{\mathrm{ij}}+\theta \cdot {\text{X}}_{\mathrm{ij}}+\varepsilon_{\mathrm{ij}}$$

where the Where *happiness*_*ij*_ is latent happiness of household *i* in community *j*, *wealth*_*ij*_ is wealth of household *i* in community *j*, *gini_j* is the Gini index in community *j*, *up*_*ij*_ is the upward wealth inequality of household *i* in community *j*, *down*_*ij*_ is the downward wealth inequality of household *i* in community *j*, test of the coefficient on *gini*_*j*_×*hage*_*ij*_ and *up*_*ij*_×*hage*_*ij*_ is used to infer moderating effect., *X*_*ij*_ is the vector of control variables, ε_*ij*_ is error item follows normal distribution.

### Results and Discussion

Table 5 represents the OLS regression results of the life cycle moderating effect on the wealth-inequality-happiness relationship. The interaction item of *gini*_*j*_×*hage*_*ij*_ is used to infer moderation.

**TABLE 5:** Moderating effect regression on happiness with the wealth Gini index throughout the life cycle.

Happiness	b	SE	t	p
**Independent variable**				
**Gini index**				
Family head’s age < 50	–0.305	0.281	–1.090	0.277
Family head’s age ≥ 50	0.404	0.265	1.530	0.127
**Gini index Family head’s age**				
Family head’s age < 50	0.006	0.007	0.830	0.404
Family head’s age ≥ 50	–0.008	0.004	–1.860	0.063

**Family covariate**				
**Youth ratio**				
Family head’s age < 50	0.231	0.059	3.910	0.000
Family head’s age ≥ 50	0.518	0.057	9.050	0.000
**Elderly ratio**				
Family head’s age < 50	0.148	0.065	2.280	0.023
Family head’s age ≥ 50	0.335	0.031	10.880	0.000
**Family’s pension**				
Family head’s age < 50	0.071	0.017	4.190	0.000
Family head’s age ≥ 50	0.123	0.016	7.540	0.000
**Family’s medicare**				
Family head’s age < 50	0.098	0.031	3.150	0.002
Family head’s age ≥ 50	0.049	0.024	2.040	0.041
**Family size**				
Family head’s age < 50	0.038	0.006	6.150	0.000
Family head’s age ≥ 50	–0.010	0.004	–2.420	0.015
**Mean of family education**				
Family head’s age < 50	0.023	0.004	5.740	0.000
Family head’s age ≥ 50	0.018	0.003	6.550	0.000
**Whether own house property**				
Family head’s age < 50	0.124	0.021	5.910	0.000
Family head’s age ≥ 50	0.067	0.017	3.960	0.000

**Family head Covariate**				
**Family head’s age**				
Family head’s age < 50	–0.009	0.004	–2.520	0.012
Family head’s age ≥ 50	0.015	0.002	6.740	0.000
**Family head’s education level**				
Family head’s age < 50	–0.002	0.003	–0.650	0.513
Family head’s age ≥ 50	0.004	0.001	2.560	0.011
**Family head’s marital status**				
Family head’s age < 50	0.266	0.021	12.630	0.000
Family head’s age ≥ 50	0.188	0.015	12.730	0.000
**Family head’s gender**				
Family head’s age < 50	0.023	0.016	1.450	0.147
Family head’s age ≥ 50	–0.026	0.012	–2.120	0.034
**Family head’s work status: Government official**				
Family head’s age < 50	0.087	0.023	3.790	0.000
Family head’s age ≥ 50	0.179	0.024	7.550	0.000
**Family head’s work status: State-owned enterprises**				
Family head’s age < 50	0.053	0.024	2.260	0.024
Family head’s age ≥ 50	0.068	0.029	2.330	0.020
**Family head’s work status: Private sector**				
Family head’s age < 50	0.062	0.015	4.020	0.000
Family head’s age ≥ 50	0.117	0.015	7.740	0.000
**Family head’s work status: Agriculture**				
Family head’s age < 50	–0.036	0.021	–1.760	0.079
Family head’s age ≥ 50	0.038	0.012	3.090	0.002
				

Regarding the life cycle effect of the family, we categorize the sample into two age groups. Empirically, the turning point of the life cycle effect on happiness usually occurs at the age of 50 (Blanchflower, 2021). The age group is classified as a younger group (age < 50) and older group (age 50). The results indicate that the older group’s inequality aversion (b = -0.008, *p* < 0.1) is more serious, while the younger group shows non-significant inequality aversion. This outcome might be the offset effect of the upward wealth inequality and the downward wealth inequality. We continue to verify how the life cycle could moderate the effect of the upward wealth inequality and the downward wealth inequality to better capture the offset effect in the Gini index.

Table 6 shows the regression of the upward wealth inequality and downward wealth on happiness in the older group and younger group. The test of the coefficient of the interaction item of *up*_*ij*_×*hage*_*ij*_ is used to infer moderation. The moderation is significantly positive, which implies that wealth inequality can influence happiness under the impact of the family life cycle. In the young-family-head group, the downward wealth inequality increases happiness significantly (b = 0.033, *p* < 0.01), whereas in the old-family-head group, the downward wealth inequality does not have a significant impact on happiness. In contrast, in the young-family-head group, upward inequality does not have a significant impact on happiness, whereas in the old-family-head group, upward inequality significantly decreases happiness (b = -1.214, *p* < 0.001).

**TABLE 6:** Moderating effect regression on happiness with the upward wealth inequality and the downward wealth inequality throughout the life cycle.

Happiness	b	SE	t	*p*
**Independent Variable**				
**The downward wealth inequality**				
Family head’s age < 50	0.033	0.013	2.650	0.008
Family head’s age ≥ 50	0.009	0.011	0.800	0.426
**The upward wealth inequality**				
Family head’s age < 50	0.076	0.225	0.340	0.736
Family head’s age ≥ 50	–1.214	0.213	–5.710	0.000
**The upward wealth inequality Family head’s age**				
Family head’s age < 50	–0.013	0.005	–2.370	0.018
Family head’s age ≥ 50	0.012	0.003	3.610	0.000

**Family covariate**				
**Youth ratio**				
Family head’s age < 50	0.223	0.059	3.800	0.000
Family head’s age ≥ 50	0.502	0.057	8.800	0.000
**Elderly ratio**				
Family head’s age < 50	0.127	0.065	1.950	0.051
Family head’s age ≥ 50	0.305	0.031	9.890	0.000
**Family’s pension**				
Family head’s age < 50	0.063	0.017	3.760	0.000
Family head’s age ≥ 50	0.110	0.016	6.730	0.000
**Family’s medicare**				
Family head’s age < 50	0.090	0.031	2.880	0.004
Family head’s age ≥ 50	0.040	0.024	1.660	0.096
**Family size**				
Family head’s age < 50	0.033	0.006	5.370	0.000
Family head’s age ≥ 50	–0.016	0.004	–3.690	0.000
**Mean of family education**				
Family head’s age < 50	0.020	0.004	4.990	0.000
Family head’s age ≥ 50	0.013	0.003	4.880	0.000
**Whether own house property**				
Family head’s age < 50	0.044	0.023	1.890	0.059
Family head’s age ≥ 50	–0.007	0.018	–0.410	0.685

**Family head Covariate**				
**Family head’s age**				
Family head’s age < 50	–0.003	0.002	–1.920	0.055
Family head’s age ≥ 50	0.008	0.001	6.820	0.000
**Family head’s education level**				
Family head’s age < 50	–0.004	0.003	–1.360	0.173
Family head’s age ≥ 50	0.002	0.001	1.090	0.277
**Family head’s marital status**				
Family head’s age < 50	0.254	0.021	12.080	0.000
Family head’s age ≥ 50	0.178	0.015	12.120	0.000
**Family head’s gender**				
Family head’s age < 50	0.017	0.016	1.110	0.269
Family head’s age ≥ 50	–0.025	0.012	–2.030	0.043
**Family head’s work status: Government official**				
Family head’s age < 50	0.084	0.023	3.660	0.000
Family head’s age ≥ 50	0.168	0.024	7.080	0.000
**Family head’s work status: State-owned enterprises**				
Family head’s age < 50	0.048	0.024	2.030	0.042
Family head’s age ≥ 50	0.055	0.029	1.890	0.058
**Family head’s work status: Private sector**				
Family head’s age < 50	0.061	0.015	4.000	0.000
Family head’s age ≥ 50	0.114	0.015	7.530	0.000
**Family head’s work status: Agriculture**				
Family head’s age < 50	–0.034	0.021	–1.630	0.102
Family head’s age ≥ 50	0.039	0.012	3.260	0.001

**FIGURE 1:**
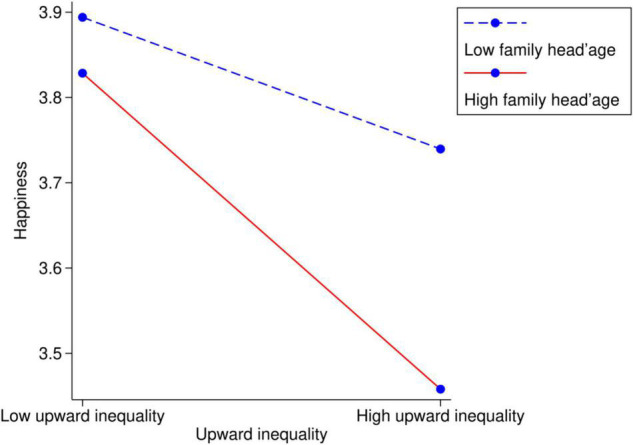
The moderating effect of family life cycle on link between the upward wealth inequality and happiness within young-family-head families (family head’s age < 50).

**FIGURE 2:**
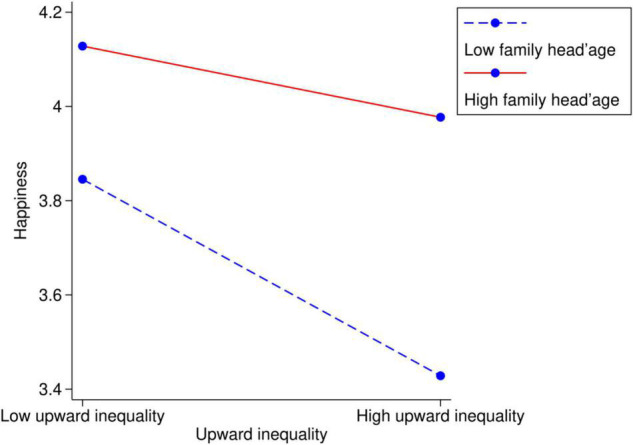
The moderating effect of family life cycle on link between the upward wealth inequality and happiness within old-family-head families (family head’s age ≥ 50).

Considering that people are more concerned with the upward wealth inequality that dominates inequality aversion, we add the interaction term *up*_*ij*_×*hage*_*ij*_ to the moderating framework. Figure 1 shows the moderating effect in the young-family-head group, and Figure 2 shows the moderating effect in the old-family-head group.

The results show that age 50 is the breakpoint of social comparison; before the family head reaches 50, the downward wealth inequality slightly increases happiness; however, after the family head reaches 50, people neglect the downward wealth inequality and focus more on the upward wealth inequality with increasing age. Furthermore, on two sides of the breakpoint, the life cycle plays different roles in the relationship between wealth inequality and happiness. As the family head approaches 50, upward inequality aversion deteriorates, while it eases as the family head goes older.

Overall, upward inequality aversion reaches a peak when the family head is approximately 50, as visualized in Figures 1, 2. At the beginning of the family life cycle, family members assume their life has limitless possibilities, and they have high expectations for the future. Logically, they can be easily satisfied by achieving a little more than their peers. In later periods, with increasing age, the members will pay more attention on health instead of wealth. For individuals in a middle-stage family, life has less potential than when they were young and is nearly unchanged. It is the moment when they look back to the past years and realize their final social status. It is the time when they care about the upward wealth inequality most.

## Discussion

The paper starts with the problem of how permanent inequality influences happiness. Then we find that the main driver of inequality aversion comes from the pain of chasing their richer peers and upward social comparison. The upward wealth inequality is characterized by a process whereby the family’s permanent economic distance with the richer ones. The empirical findings from this paper support that inequality aversion comes from social comparison, specifically, it comes from upward social comparison. As shown in the empirical test of hypothesis 2, unlike urban resident, families who live in rural areas do not have significant proud effect. Moreover, the empirical test of hypothesis 3 implies that during the family life cycle, inequality aversion will be different in different life stages due to the changes in economic status expectations.

Unlike the temporary inequality aversion, the permanent inequality aversion comes from desire of higher economic status. Cojocaru (2014) reveals that income inequality aversion, which is temporary inequality, is morally objectionable. Cojocaru’s study shows that temporary inequality aversion is tied to concerns with fairness. Thus, both upward wealth inequality and downward wealth inequality are negatively associated with happiness. However, the evidence in this paper indicates that permanent inequality is less associated with unfairness but more sensitive to the economic status in the reference group. If they are relatively high -status, they are happier.

In the context of rapid urbanization, the urban-rural gap is increasing. Even the rich in rural areas still the lower classes in the whole society, let alone the others. They feel no sense of achievement but the deprivation from the economic growth. China is fairly representative of other developing countries, which are also experiencing rapid economic growth and urbanization. Even though the economy develops, the negative effects of urbanization should also get noticed by the government. The government should take actions like improving the construction of digital finance, transportation and education to enhance the economic situation of rural residents (Li et al., 2021).

the effects of moderating of life cycle show the upward wealth inequality aversion weakens across the life span of family, peaking around the middle stage (family head’s age is around 50) of family life span and decreasing from middle stage to older stage. According to our results, the key aspect of correcting for the negative effects of unequal preparation is focusing on wealth inequality within families of the middle-stage phase of the life cycle (family head’s age is around 50). The upward wealth inequality aversion is strongest among those families. It is due to the change of expected age-graded pattern of potential and the extent of the interpersonal contact. The earlier research shows the prospect develops during the life span (Heckhausen and Baltes, 1991; Heckhausen and Krueger, 1993). From the early adulthood to the old age, the expected economic status shows a gradual shift from the predominance of gains toward a predominance of losses. From the beginning to the middle-stage, the expected age-graded pattern of the family decreases and it aggravates the upward wealth inequality aversion since they realize the gap between them and the richer ones is harder and harder to close as they approach the middle-stage phase. The upward wealth inequality aversion is strongest around the middle-stage phase of the family. After that, from middle-stage phase to latter-stage phase, the family members in the older family tend to engage less in social comparison due to the losses of interpersonal comparison and declines in the cognitive capacities required in social comparison or they just focus on the health instead of economic status.

The middle-stage targeting policy not only could modify the middle-stage families but also benefit families of all stages, which explains why the promoting equality policy should be targeted at the middle-stage phase of families. The young families could be more anxious about the upward wealth inequality if they observe the middle-stage families still face the severe inequality and they might struggle harder to catch up with the Joneses. The excessive competition of young families harms social welfare. On the contrary, this policy relieves the young families’ inequality aversion by charting a more equal prospect in their later years and it naturally reduces the inequality for older families.

This study has several issues that need to be acknowledged. Firstly, we empirically tested the study using cross-sectional data. Secondly, we use the self-reported happiness as the measure of happiness. Nevertheless, the self-reported happiness could be the instant state of mind when the survey holds. Future research can examine the relationship between using daily data, if available, to link the variations in daily happiness to the permanent inequality. It could change easily. Thirdly, this paper chooses the family in the same community as the reference group, however, one possibility is that one’s self-evaluation might more rely on one’s personal past than others (Almond, 2015). The choice of reference group might vary with families, the involving survey needs to be conducted. Lack of information, this paper chooses the community as the reference group considering the geographical factors. Notwithstanding, future research should consider ask the exact reference group during the survey.

Our findings shed light on the pathway by which macroeconomic inequality affects the psychological movement of individuals. Moreover, mental activities may further influence individual economic behavior. The powerful motivator to promote economic status aroused by the upward wealth inequality might increase conspicuous consumption, especially among young people and the poor. When they are lack money, the desire for unrealistic status could easily get them to over-borrowing. Because conspicuous consumption creates a mismatch between the income and spending level for those low-income groups. Conspicuous consumption worsens the upward gap for them. The upward wealth inequality and conspicuous consumption create a vicious cycle. Thereby, both the mechanism of this vicious cycle and other permanent inequality influences that cause people to take extreme a should be taken into consideration by researchers in the future.

## Data Availability Statement

Publicly available datasets were analyzed in this study. The datasets for this study can be found in the Survey and Research Center for China Household Finance, available at: https://chfs.swufe.edu.cn/.

## Author Contributions

LG and BS codesigned the study. BS interpreted the data. LG drafted the manuscript. ZD analyzed the data. GL, LG, BS, and ZD revised the manuscript. All authors contributed to the article and approved the submitted version.

## Conflict of Interest

The authors declare that the research was conducted in the absence of any commercial or financial relationships that could be construed as a potential conflict of interest.

## Publisher’s Note

All claims expressed in this article are solely those of the authors and do not necessarily represent those of their affiliated organizations, or those of the publisher, the editors and the reviewers. Any product that may be evaluated in this article, or claim that may be made by its manufacturer, is not guaranteed or endorsed by the publisher.
